# Small cell neuroendocrine tumor of the breast in a 40 year-old woman: a case report

**DOI:** 10.1186/1752-1947-4-201

**Published:** 2010-06-30

**Authors:** Stefania Nicoletti, Maximilian Papi, Fabrizio Drudi, Manuela Fantini, Debora Canuti, Emiliano Tamburini, Cinzia Possenti, Enzo Pasquini, Massimo Brisigotti, Alberto Ravaioli

**Affiliations:** 1 Oncology and Oncoematology Department, 'Infermi' Hospital, Via Settembrini 2, Rimini, 47921 Italy; 2 Oncology Department, 'Cervesi' Hospital, Via Ludwig Van Beethoven, Cattolica, 47841 Italy; 3 IRST (The Cancer Institute of Romagna), Via Piero Maroncelli 40, Meldola (FC) 47014, Italy

## Abstract

**Introduction:**

Small cell neuroendocrine cancer of the breast is a rare tumor with less than 30 cases reported in the literature. The morphological and immunohistochemical patterns of this tumor are similar to small cell neuroendocrine cancer of the lung. For this reason, it is often difficult to distinguish a primary small cell neuroendocrine cancer of the breast from a metastatic lesion from other sites.

**Case presentation:**

We report and characterize with immunohistochemical techniques a case of primary small cell neuroendocrine cancer of the breast occurring in a 40-year-old Caucasian woman. A palpable and mobile 3.0 cm tumor was located in the upper-outer quadrant of her right breast. Lumpectomy and subsequent radical mastectomy with axillary lymph node resection were performed. Microscopically, the tumor consisted predominantly of a diffuse proliferation of small oat cells. The tumor cells were positive for neuroendocrine markers chromogranin A and synaptophysin. One of 16 lymph nodes was metastatic. A correct treatment needs to be chosen.

**Conclusions:**

It has recently been demonstrated that early small cell neuroendocrine cancer of the breast shows a good prognosis with adjuvant treatments with high disease free survival. Our patient is alive and well without disease eight years after treatment. We performed an adjuvant therapy with the classic scheme doxorubicin and cyclophosphamide, followed by carboplatin and etoposide. A more extensive review is required to define a standard treatment protocol for this rare neoplasm.

## Introduction

Neuroendocrine (NE) carcinomas of the breast are defined by the diffuse expression of NE markers (chromogranins and/or synaptophysin) in ≥50% of cells. This definition includes lesions with pure NE phenotype as well as variants which may co-express mucinous and/or apocrine phenotype. The existence of primary breast carcinoid tumors is still controversial and, if accepted, it would account for less than 1% of primary breast cancers [1]. In this report, we describe a case of primary small cell neuroendocrine carcinoma (SCNC) of the breast occurring in a 40-year-old woman.

## Case presentation

**Figure 1:**
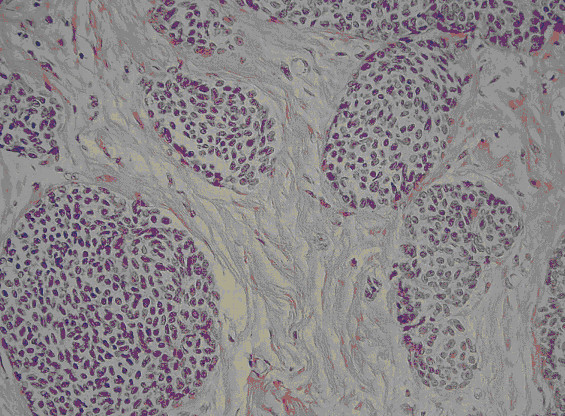
**Histopathologic characteristics of the tumor**.

In February 2000, a 40-year-old Caucasian woman developed a palpable mobile node in her right breast, 3.0 cm in diameter. The mass was located in the upper-outer quadrant of her right breast. Mammography and ecography revealed the nodule to have suspicious characteristics. The biopsy of the lump revealed a SCNC of the breast. The personal history of our patient was characterized by first degree family history of breast cancer. Her mother was diagnosed with breast cancer at 57 years old and her mother's aunt with SCNC of the lung at 73 years of age. At the time of evaluation, our patient was in good general condition, without other co-morbidities. She was treated with mastectomy and axillary lymph node resection. Our patient was in good health with a Performance Status 0 (ECOG), with negative routine laboratory investigations, normal serum breast cancer markers and chromogranin of 45 ng/mL. A thorough examination (abdominal ultrasound, total-body computerized tomography, bone scintigraphy, pelvic and transvaginal ultrasound) showed no evidence of metastases. Macroscopically, the tumor was 3 cm in maximum diameter (pT2). It was yellowish-white with large areas of coagulative necrosis, ductal hyperplasia and fibrous parenchyma. Widespread vascular invasion was present. One of 16 lymph nodes was metastatic (pN1a). Microscopically, the tumor was characterized by atypical cells with dimorphic nuclei and scant cytoplasm, organized in solid and trabecular arrangements. Widespread necrosis was present. The tumor cells were highly positive for neuron specific enolase (NSE), chromogranin and synaptophysin and negative for c-erb-B2 and cytocheratin 20 (Figure 1). Estrogen receptors were positive in 80% of the tumor cells, progesterone receptors were positive in 90%, Ki-67 90%, c-erb-B2 absent, p53 90%. No ductal carcinoma *in situ *was observed. For this reason, we obtained two separate revisions of the samples, one by the Pathology Department of "M. Malpighi" Hospital in Bologna and one by the Pathology Department of the Oncologic Institute in Milan (IEO) confirmed the diagnosis.

With immunohistochemical analysis, a distinction between a primary tumor and a metastatic lesion is possible: SCNC of the breast is positive for cytocheratin 7 and negative for cytocheratin 20, whereas the SCNC of the lung is negative for both. SCNC of the breast can be confused also with a lobular breast cancer. Generally, in SCNC an electron microscopy will show neurosecretory granules, which are reminiscent of the argyrophil variant of lobular carcinoma of the breast. Lobular carcinoma is always negative for Caderin E, whereas SCNC is positive in 100% of cases as in this situation.

Our patient was treated with chemotherapy, doxorubicin 60 mg/m^2 ^and cyclophosphamide 600 mg/m^2 ^(AC) with four cycles every 21 days, followed by carboplatin 300 mg/m^2 ^during day one and etoposide 120 mg/m^2 ^during days one to three, (CE) for three cycles every 28 days. These drugs were chosen for their described efficacy both in breast carcinoma as in SCLC. Cisplatin or carboplatin with etoposide are considered standard treatments in SCLC [2-4], while doxorubicin is the most used drug for breast carcinoma and is also active in SCLS. Hematological and clinical compliance was poor with main toxicities being neutropenia G3 and fatigue G2. No major complications were observed and our patient completed treatment at the full dosage. In consideration of the positive hormonal status and of the negative c-erb-B2, after chemotherapy, our patient received tamoxifen 20 mg daily for five years plus LH-RH analogues for two years. Our patient underwent a regular follow-up check-up every six months. After 18 months, tamoxifen was discontinued for intolerance and minor vascular complications, and was replaced with anastrozole from November 2005. At that time, our patient became amenorrhoic and follicle-stimulating hormone (FSH) and luteal hormone (LH) were compatible with menopausal status. Our patient is alive and well without disease after eight years.

## Discussion

Small cell (oat cell) neuroendocrine cancer of the breast is a rare tumor with fewer than 30 cases reported in the literature. The morphological and immunohistochemical patterns of this tumor are similar to SCNC of the lung [5]. For this reason, it is often difficult to distinguish a primary SCNC of the breast from a metastatic lesion from other sites [6-8]. Literature shows very poor prognosis for the SCNC of the breast without adjuvant treatment. These tumors often arise with extensive vascular invasion, nodal involvement, high proliferation index and more frequently, without estrogen and progesterone expression [9-11]. Mucin production is a common feature in NE breast tumor and the mucinous differentiation is an important indicator of low biological aggressiveness. Estrogen and progesterone expression is also correlated with a better prognosis [1].

Wade *et al*. described in 1983 the first SCNC of the breast. Another seven cases were described in the period 1983 to 1995 with extremely poor prognoses (median survival nine to ten months). Currently, SCNC of the breast is considered an extremely aggressive tumor for which there is no general agreement about a standardized treatment [12,13]. The best choice seems to be radical mastectomy with axillary resection followed by a chemotherapy combining anthracycline and effective drugs for small call carcinoma (platinum compounds and etoposide). We adopted this therapeutic choice for our patient, adding hormonal treatment at the light of the positive hormonal receptor status. Our patient is still alive and well without disease eight years after surgery and medical treatment.

## Conclusions

Small cell (oat cell) neuroendocrine cancer of the breast is a rare tumor. It has been recently demonstrated that early SCNC of the breast shows a good response to adjuvant treatment with a disease free survival ranging between 33 and 48 months. Further studies with more cases are required to define more precisely treatment indications for this rare neoplasm.

## Consent

Written informed consent was obtained from our patient for publication of this case report and any accompanying images. A copy of the written consent is available for review by the Editor-in-Chief of this journal.

## Competing interests

The authors declare that they have no competing interests.

## Authors' contributions

SN, FD, MF, ET and AR described and wrote the case report. MB performed the pathological analysis; MP, DC and CP revised literature. EP performed the last revision. All authors have read and approved the final manuscript.
